# Ventral Striatal Activity Correlates with Memory Confidence for Old- and New-Responses in a Difficult Recognition Test

**DOI:** 10.1371/journal.pone.0054324

**Published:** 2013-03-05

**Authors:** Ulrike Schwarze, Ulrike Bingel, David Badre, Tobias Sommer

**Affiliations:** 1 Department of Systems Neuroscience, University Medical Center Hamburg-Eppendorf, Hamburg, Germany; 2 Department of Neurology, University Medical Center Hamburg-Eppendorf, Hamburg, Germany; 3 Department of Cognitive, Linguistic and Psychological Sciences, Brown University, Providence, Rhode Island, United States of America; Centre Hospitalier Universitaire Vaudois Lausanne - CHUV, UNIL, Switzerland

## Abstract

Activity in the ventral striatum has frequently been associated with retrieval success, i.e., it is higher for hits than correct rejections. Based on the prominent role of the ventral striatum in the reward circuit, its activity has been interpreted to reflect the higher subjective value of hits compared to correct rejections in standard recognition tests. This hypothesis was supported by a recent study showing that ventral striatal activity is higher for correct rejections than hits when the value of rejections is increased by external incentives. These findings imply that the striatal response during recognition is context-sensitive and modulated by the adaptive significance of “oldness” or “newness” to the current goals. The present study is based on the idea that not only external incentives, but also other deviations from standard recognition tests which affect the subjective value of specific response types should modulate striatal activity. Therefore, we explored ventral striatal activity in an unusually difficult recognition test that was characterized by low levels of confidence and accuracy. Based on the human uncertainty aversion, in such a recognition context, the subjective value of all high confident decisions is expected to be higher than usual, i.e., also rejecting items with high certainty is deemed rewarding. In an accompanying behavioural experiment, participants rated the pleasantness of each recognition response. As hypothesized, ventral striatal activity correlated in the current unusually difficult recognition test not only with retrieval success, but also with confidence. Moreover, participants indicated that they were more satisfied by higher confidence in addition to perceived oldness of an item. Taken together, the results are in line with the hypothesis that ventral striatal activity during recognition codes the subjective value of different response types that is modulated by the context of the recognition test.

## Introduction

Successful recognition is supported by a large network of brain regions including medial temporal, prefrontal and parietal areas. Activity in the ventral striatum (VS) is also associated with retrieval success, i.e. VS activity is higher for correctly recognized old items than for correctly rejected new items [1]–[4]. However, the functional significance of ventral striatal activity during recognition has so far received little scientific attention. This area has usually been studied as a central part of the reward circuit, which is involved in reinforcement learning and motivational behaviour [5]. In addition, the VS is associated with the processing of salience and novelty [6], [7].

Based on the prominent role of the VS in the reward circuit, it has been speculated that its retrieval success activity reflects that humans value hits more than correct rejections in a standard recognition memory test [1]. Only recently has a study directly examined the role of the VS in recognition memory [8]. The authors investigated whether VS activity signals successful retrieval triggered by hippocampal and thalamic regions (‘retrieval-dependent account’) or whether VS activity reflects a subjective preference for detecting a previously studied item (‘goal-dependent account’). After studying word lists, subjects were cued whether hits or correct rejections were rewarded in the subsequent recognition test-phase in order to manipulate the motivational status of ‘old’- and ‘new’-responses. In both conditions, responses concordant with the incentive manipulation led to increased striatal activity, i.e. ‘new’-responses were associated with striatal activity when correct rejections were externally incentivized and ‘old’-responses were associated with VS activity when hits were rewarded [8].

This pattern of results implies that striatal activity during recognition is the result of an active evaluation process rather than an automatic consequence of memory reactivation. Thus, it corroborates the goal-dependent account. Especially the unusual activity elicited by ‘new’-responses (when rewarding correct rejections) implicates that the striatal response during recognition is context-sensitive and modulated by the adaptive significance of “oldness” or “newness” to the current goals [4]. This conclusion is supported by enhanced striatal activity for novel items observed in other, non-recognition memory contexts, since such a novelty response would be not consistent with an automatic striatal response to perceived oldness [6], [9]. In summary, the VS seems to assign a subjective value to the degree of familiarity of an item that depends on the current behavioural goals [4].

The goal-dependent hypothesis suggests that also other deviations from standard recognition tests that affect the subjective value of specific response types should modulate the VS activity pattern. In the current study, we explored VS activity in an unusually difficult recognition test that was characterized by very low levels of confidence and accuracy. Based on the human uncertainty aversion [10], [11], the subjective value of all high confident decisions is expected to be higher than usual in such a recognition context, i.e. also rejecting items with high certainty is evaluated as concordant with the current goals. Accordingly, one would hypothesize that VS activity in such a recognition test is not only correlated with retrieval success, but in addition with mnestic confidence not only for ‘old’- but also and ‘new’-responses. On the other hand, when mnestic confidence was previously investigated using standard recognition tests with relatively high certainty, activity in the ventral, reward-associated part of the striatum was not reported by the vast majority of studies – with the exception of one study that observed a small cluster of superthreshold voxels [12]–[18]. Therefore, the VS seems not to be an obligatory node in the confidence network for recognition tasks with the usual high rate of high confidence responses. Moreover, these studies - with one exception - analysed only activity associated with correct ‘old’-responses, i.e. hits, of varying confidence [17]. For this reason, their findings cannot be generalized to the neural correlates of confidence for ‘new’- and incorrect responses. In summary, the present study aimed at exploring the hypothesis that high uncertainty during recognition leads - in contrast to standard paradigms - to VS activity correlated with mnestic confidence for both ‘old’- and ‘new’-responses. To validate that VS activity is, as hypothesized, related to the subjective value of different retrieval outcomes, participants rated in an accompanying behavioural experiment the pleasantness of each recognition response. This procedure avoids reverse inference from VS activity to the subjective value of the various confidence levels [19].

The unusually high false alarm and miss rates in the present recognition paradigm provided the opportunity to test, as a subordinate hypothesis, whether the correlation of retrieval success with VS activity is influenced by the correctness of ‘old’- and ‘new’-decisions. According to the goal-dependent account, the *subjective perception of oldness* (‘perceived oldness’) [20] should correlate with VS activity independent of the factual status of an item, i.e. hits or false alarm [4].

## Materials and Methods

### Experiment 1

#### Experimental paradigm

For the fMRI study, 19 volunteers (5 female, age 27.3±3.3 years) encoded 80 unfamiliar photos of outdoor scenes [21], which were presented only briefly followed by an active baseline condition (800 ms, ISI 8–12 s, arrow pointing task). During encoding, subjects indicated the category of each picture, i.e. whether it contained cars or people, via button press. Half of all pictures were followed by a brief nociceptive stimulus, i.e. electrical shock. This encoding manipulation is not relevant for the present report and its details as well as its influence on the neural correlates of encoding are reported elsewhere [22]. However, all fMRI and behavioural analyses of the current report were controlled for potential effects of nociceptive stimulation on performance and activity during recognition.

During recognition on the following day, all pictures were presented together with the same amount of lures. For each photo (presented for 6 s, ISI 3–6 s), subjects indicated their memory confidence on a 6-point confidence scale (1- “high confidence old”, 6 - “high confidence new”) using an MRI-compatible computer mouse. Feedback about the correctness of the response was not given. Brain activity was measured using fMRI during encoding and recognition.

#### Image acquisition and pre-processing

FMRI was performed on a 3T system (Siemens Trio) with an EPI T2*-sensitive sequence in 40 contiguous axial slices (2 mm thickness with 1 mm gap, TR 2.38 s). The imaging series were pre-processed using SPM8 (http://www.fil.ion.ucl.ac.uk/spm/), i.e. slice-time corrected, realigned and corrected for the interaction of motion and distortion, spatially normalized into standard anatomical space (MNI), and smoothed with a Gaussian kernel of 8 mm fwhm. Univariate single subject (first-level) and group (second-level) statistics were conducted using the general linear model implemented in SPM8.

fMRI analyses. The rationale for the series of fMRI analyses was to first test the primary hypothesis, i.e. that striatal activity in a difficult recognition test is correlated not only with perceived oldness but also with confidence. The result of this primary analysis was then corroborated by three complementary analyses in order to explore i) whether the correlation of VS activity and confidence exists for ‘old’- and ‘new’-responses, i.e. is irrespective of perceived oldness, ii) whether the correlation of VS activity with confidence can be found for correct and incorrect responses, i.e. irrespective of the factual status of an item, and iii) to characterize the exact relationship between confidence levels as well as perceived oldness/newness and VS activity. The subordinate hypothesis, that perceived oldness and not only hits are correlated with VS activity, was tested using the second complementary model.

In order to test the primary hypothesis and to identify areas where activity correlates not only with perceived oldness but also confidence, both variables were included in a first-level model as parametric regressors. In particular, delta functions marking trial-onsets of all 160 recognition events were convolved with the canonical hemodynamic response function to create an event-related regressor. This onset regressor was modulated by 8 parametric regressors. The first 6 parametric regressors were introduced to account for variance of no primary interest: While the first three parametric regressors coded for the electric shock during encoding and its interaction with confidence and perceived oldness, the following three regressors coded for correctness during recognition and its interaction with confidence and perceived oldness. The final two parametric regressors coded for the variables of interest, i.e. perceived oldness (new = −1, old = 1) and confidence (low = −1, medium = 0, high = 1).

The order of parametric regressors was chosen to remove all variance explained by the variables of no primary interest, i.e. the electric shock during encoding and correctness, by the serial orthogonalization of parametric regressors as implemented in SPM8. In order to test for the validity of the subsequent analyses, the co-linearity of the parametric regressors coding for perceived oldness and confidence was computed without orthogonalization. The two regressors were found to be non-colinear (mean cosine 0.06) and therefore did not affect each other by the serial orthogonalization procedure prior to model estimation.

For the second-level model, the parameter estimates of the confidence and the perceived oldness parametric regressors were included treating subjects as random effects. First, one sample t-tests were conducted to identify areas where activity correlates with confidence and perceived oldness, respectively. Second, a conjunction analysis was conducted to identify areas where activity correlated with both processes. In order to identify areas where activity correlated with one of the two processes, the particular t-map was exclusively masked by the other contrast with a threshold of p<0.05 uncorrected. The conjunction and masking analyses were interpretable since both regressors were orthogonal and therefore unchanged by the serial orthogonalization. The masking analyses were corroborated by statistically contrasting the activity associated with perceived oldness and confidence. This statistical comparison of parametric regressors was valid in the current analysis since both regressors were not only orthogonal but also scaled identically from −1 to +1 and assigned each trial to the discrete levels of a 2 (old/new) ×3 (confidence) factorial design.

The first complementary analysis was conducted to confirm that the correlation of VS activity with confidence holds true for ‘old’- and ‘new’-responses, i.e. is irrespective of perceived oldness. In the first-level model, two onset regressors coded for ‘old’- and ‘new’-responses. These onset regressors were again modulated by parametric regressors to account for variance of no primary interest, i.e. the electric shock and its interactions and correctness. The last parametric regressor of both onset regressors coded for confidence. On the second-level, a conjunction analysis of the parameter estimates of these confidence regressors was computed to identify areas where activity correlated with confidence for ‘old’- as well as ‘new’-responses.

The second complementary analysis was conducted to explore whether striatal activity associated with confidence is independent of the factual status of an item, i.e. whether it was a target or a lure. In the corresponding first-level model, two onset regressors coded for correct and incorrect responses. As before, the first three parametric regressors accounted for variance of no primary interest. The fourth regressor coded for perceived oldness, i.e. hits vs. correct rejections and false alarms vs. misses. The last parametric regressor of both onset regressors coded for confidence. On the second-level, a conjunction analysis of the parameter estimates of these regressors was computed to identify areas where activity correlated with the confidence of an item irrespective of the correctness of the decision. A conjunction analysis on the second level of the two regressors coding for perceived oldness allowed us to test the subordinate hypothesis that perceived and not factual oldness is associated with VS activity.

The third complementary analysis was conducted to characterize the exact shape of the relationship between VS activity and confidence since the parametric regressors coding for confidence test specifically for a linear function. Therefore, a first-level analysis was conducted in which each of the six confidence levels was modelled as a separate onset regressor. This model allowed us to compute the activity associated with the various confidence levels in voxels identified by the primary model.

For all analyses, the threshold was set to p<0.05 corrected for multiple comparisons within regions of interest (ROIs). Based on the a priori hypothesis, the present study focuses on the VS and regions that were previously identified as nodes of a ‘confidence’ network. An anatomical mask of the nucleus accumbens/VS was created using the Harvard-Oxford cortical and subcortical structural atlases as implemented in FSL (www.fmrib.ox.ac.uk/fsl). Regions of the ‘confidence’ network during recognition included hippocampus, parahippocampus, putamen, inferior frontal gyrus, cingulate cortices, and cunei [17]. The search volumes for these ROIs were defined as spheres of 10 mm radius centred around the previously reported peaks of activity. Small volume correction was done separately within each ROI.

### Experiment 2

In order to assess the subjective value of the six confidence levels during recognition, 19 new participants (9 female, age 25.6±2.9 years) were tested with a modified version of the same recognition paradigm outside of the MR scanner. Stimulus material, instructions and timing were identical, only the electrical stimulation during encoding was omitted since it was not relevant for the second experiment and did not affect recognition performance. During recognition, a visual analogue scale (VAS; which ranged from ‘very unpleasant’ to ‘very pleasant’) was presented after each confidence rating. A VAS was chosen to minimize an implicit transfer from the confidence to the pleasantness ratings. Participants were asked to indicate how much the last response satisfied them by compressing or expanding the scale. They were instructed to use their subjective criteria for this decision and were asked about their criteria at the end of the experiment.

## Results

### Behavioural Results

#### Memory performance

For Experiment 1, the analysis of the behavioural data revealed that neither correctness nor confidence during recognition were influenced by the encoding manipulation(accuracy F(1,18) = 0.3, n.s.; confidence F(1.9,35.9) = 1.4, n.s.) [22]. Therefore, memory performance for this group was pooled across encoding conditions. For both, Experiment 1 and 2, recognition performance was very low (see Table 1). But, the corrected hit (hit – false alarms of the same confidence level) and corrected rejection (correct rejections – misses of the same confidence level) rates were above chance (with the exception of low confidence hits, experiment 1: t(18) = 1.1, n.s., experiment 2: t(18) = 1.9, n.s.). High confidence responses were significantly less frequent than the other two confidence ranks (experiment 1: F(2,36; Greenhouse-Geisser (GG) 1.5,26,8) = 12.4, p<0.05, experiment 2: F(2,36; GG 1.4,25.9) = 17.4, p<0.05, Bonferroni pairwise comparisons p<0.05). Targets and lures were experienced as highly similar, indicated by a d-prime of 0.61 and 0.62 (response criterion 0.14 and 0.18, for Experiment 1 and Experiment 2 respectively).

**Table 1:** Descriptive statistics of the behavioural data (Proportion of all trials, standard deviation in parentheses, confidence ratings: 1: high confidence old, 6: high confidence new).

	*1*	*2*	*3*	*4*	*5*	*6*
Study I						
Old items	16.7 (11.0)	16.8 (7.9)	20.9 (9.9)	21.3 (6.9)	14.7 (7.4)	5.8 (8.3)
New items	4.1 (4.5)	10.4 (5.2)	19.3 (9.6)	27.7 (9.7)	23.3 (10.6)	12.5 (14.4)
Study II						
Old items	17.1 (11.9)	15.9 (8.9)	21.8(10.8)	20.2 (9.0)	16.8 (12.1)	4.2 (4.1)
New items	3.6 (4.9)	10.6 (6.2)	18.2 (9.9)	28.8 (11.2)	25.8 (10.7)	8.3 (7.2)

* the proportions for both old and new items do not sum up to 100 due to missing responses in the time window of 6 s.

#### Pleasantness ratings

**Figure 1:**
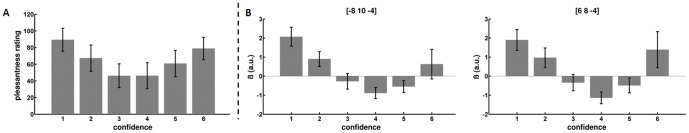
Activity in the ventral striatum (experiment 1) and pleasantness ratings (experiment 2). A: Pleasantness ratings (mean±SD) for all confidence levels (1 = high confidence old, 6 = high confidence new), irrespective of correctness. B: Parameter estimates of the third complementary analysis (mean±SEM, in arbitrary units) for all confidence levels (1 = high confidence old, 6 = high confidence new), irrespective of correctness in the peak voxels of the primary conjunction analysis at xyz = [−8 10 −4] and [6 8 −4]). For details of the analyses see the main text.

The mean pleasantness ratings for different confidence levels are plotted in Figure 1A. A repeated measure ANOVA with the factors ‘old/new-response’ and ‘confidence’ revealed significant main and interaction effects (main effect old/new: F(1,18) = 10.49, p = 0.004; main effect confidence: F(2,36; GG 1.29,23.3) = 62.17, p<0.001; interaction old/new x confidence: F(2,36; GG 1.4, 25.9) = 7.54, p = 0.001). When asked for the criteria for the pleasantness ratings at the end of the experiment, 14 out of 19 participants reported that pleasantness referred to subjective confidence in a given trial. For most of them (n = 9), perceived oldness was a second, subordinated criterion. In contrast, 2 participants reported that they judged old-responses more pleasant than new responses, irrespective of confidence. Two participants were satisfied when their classification matched their feeling about an item. One participant could not provide a precise rule for his pleasantness ratings.

### Functional Results

**Figure 2:**
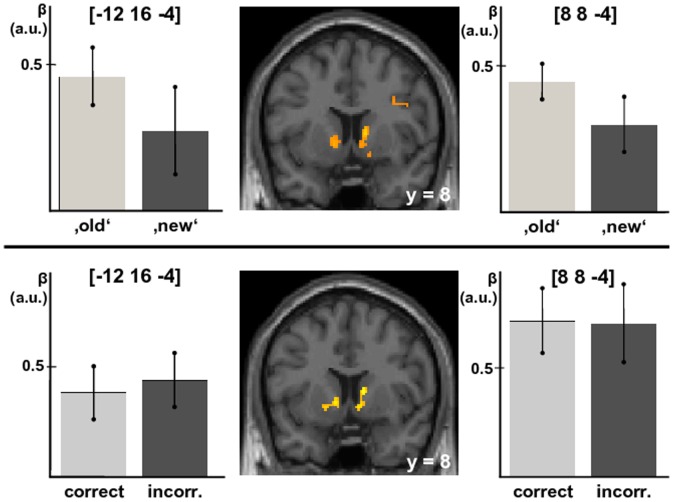
Ventral striatal activity related to perceived oldness (p.o.) and confidence (c.). Red clusters represent the results of the conjunction analysis; yellow clusters represent increased striatal activity due to increasing confidence exclusively masked by perceived oldness. The bars represent the parameter estimates (β; mean±SEM, in arbitrary units) of the corresponding parametric regressors (see text for details of the analysis). For visualization purposes, a threshold of p<0.001 uncorrected was applied.

The primary analysis revealed that activity correlated with confidence in areas of the ‘confidence-network’: the left angular gyrus, bilateral posterior and anterior cingulate cortices, cunei, inferior frontal gyri as well as hippocampi [17]. Crucially, in the current study, this ‘confidence-network’ was extended to include the bilateral VS (left VS: peak voxel coordinates: xyz = [−8 10 −4], Z-value of peak voxel = 5.43, p<0.001 adjusted for small volume correction (svc.); right VS: peak voxel xyz = [6 8 −4], Z = 5.14, p<0.001 svc; Figure 2).

**Figure 3:**
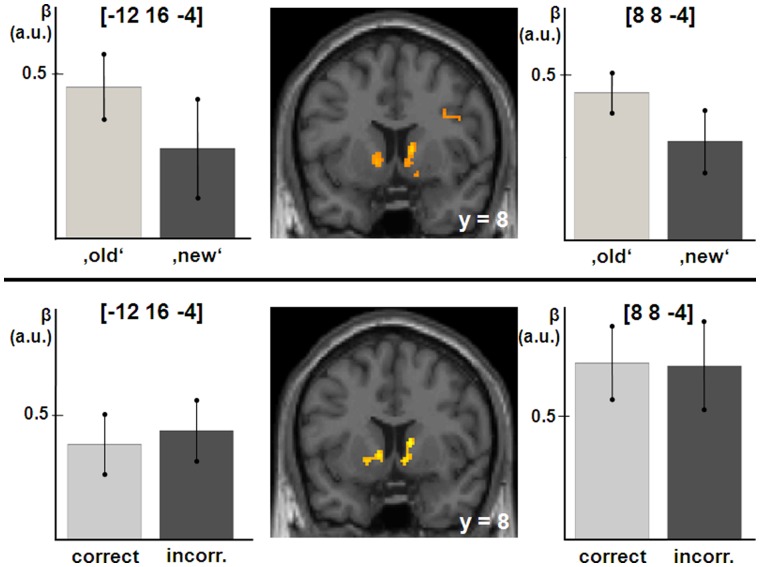
Top: Ventral striatal activity related to confidence for perceived ‚old‘- and ‚new’-responses, i.e. hits and false alarms, correct rejections and misses; Bottom: Ventral striatal activity related to confidence for correct and incorrect responses, i.e. hits and correct rejections, false alarms and misses. The bars represent the parameter estimates (β; mean±SEM, in arbitrary units) of the corresponding parametric regressors in the peak voxel named above the bars. For visualization purposes, a threshold of p<0.001 uncorrected was applied.

The first complementary analysis confirmed that activity correlated with confidence for both ‘old’- and ‘new’-decisions (left VS peak voxel xyz = [−12 16 −4], Z = 3.36, p = 0.006 svc.; right VS peak voxels xyz = [8 8 −4], Z = 3.26 p = 0.008 svc.; xyz = [12 10 −10], Z = 3.23 p = 0.009 svc.; Figure 3 top). Moreover, this correlation between confidence and VS activity was evident independent of the correctness of the response, as revealed by the second complementary analysis (left VS peak xyz = [−12 16 −4], Z = 3.62, p = 0.004 svc.; right VS peak voxel xyz = [−12 16 −4], Z = 3.82, p = 0.002, svc.; Figure 3 bottom).

Bilateral VS activity was also correlated with perceived oldness, i.e. activity was greater during ‘old’- than ‘new’-decisions (same peak voxels as in the ‘confidence’ analysis; left VS: peak voxel xyz = [−8 10 −4]; Z = 5.03, p<0.001, svc.; right VS: peak voxel xyz = [6 8 −4]; Z = 3.92, p = 0.001, svc.). The second complementary analysis revealed that this enhanced activity for perceived oldness was independent of the factual status of an item, i.e. VS activity was greater for hits than correct rejections and for false alarms than misses (left VS: xyz = [−8 10 −4], Z = 4.15, p<0.001 svc; right VS: xyz = [10 14 −4], Z = 2.88, p<0.025 svc).

The conjunction analysis of confidence and perceived oldness revealed that activity in overlapping areas of the VS correlate with both confidence and perceived oldness (left VS: peak voxel xyz = [−8 10 −4], Z = 5.03, p<0.001, svc.; right VS: peak voxel xyz = [6 8 −4], Z = 3.92, p = 0.001, svc.; Figure 2). Exclusive masking revealed that activity in more right ventral and bilateral anterior areas of the VS correlated significantly only with confidence but not with perceived oldness (left anterior VS: peak voxel xyz = [−12 18 −6]; Z = 4.2, p<0.001, svc.; right inferior ventral VS: peak voxel xyz = [10 10 −10], Z = 4.55, p<0.001, svc.; right anterior VS: peak voxel xyz = [12 16 −8], Z = 3.18, p = 0.01, svc.; Figure 2). This descriptive activity difference was corroborated statistically and the differential contrast, i.e. confidence +1 and perceived oldness −1, revealed a cluster in the right ventral VS (peak voxel: xyz = [12 10 −10]; Z = 2.77, p<0.05, svc.). No significant activation was found in the reverse masking and differential contrast analyses.

The results of the third complementary analysis to characterize the relationship between confidence and VS activity are plotted in Figure 3B and suggest a linear response function. A perceived oldness/newness x confidence ANOVA revealed for the right and left VS significant effects of perceived oldness/newness and confidence (right VS: old/new F(1,17) = 7.47, p = 0.01, confidence F(2,34; 1.63,27.72) = 13.11, p<0.001; left VS: old/new F(1,17) = 16.05, p<0.001, confidence F(2,34; GG 1.61,27.38) = 9.49, p<0.001). This ANOVA confirmed therefore the results of the primary analysis.

## Discussion

VS activity correlated in the current unusually difficult recognition test not only with retrieval success, but also with confidence. Moreover, consistent with the well described uncertainty aversion [10], [11], participants indicted in the behavioural experiment that they were more satisfied by higher confidence in addition to perceived oldness, which parallels the VS activity pattern. In contrast, the vast majority of previous item-memory studies on the neural correlates of recognition confidence did not observe VS activity above the statistical threshold [12]–[18]. Only two difficult source memory studies reported larger clusters of VS activity to be associated with confidence [23], [24]. This across study pattern implies that the VS is not an obligatory node of the ‘confidence’-network, as e.g. parietal areas, but that its correlation with confidence might be a function of task difficulty. Therefore, it is plausible to attribute its correlation with confidence in the present study to the unusual task difficulty that resulted in high subjective values of all high confident retrieval outcomes. The observed context sensitivity of the striatal signal parallels the findings of Han and colleagues who increased the subjective value of correct rejections by external incentives. Taken together, the results are therefore in line with the hypothesis that VS activity during recognition is not an automatic result of memory reactivation, but codes the subjective value of different response types that is modulated by context [4], [8].

In the decision making literature, the high subjective value of certain information in an uncertain environment was recently illustrated by the willingness to pay for information [25]. The resulting increase in decision confidence correlated with VS activity and even certain information about upcoming punishments activated the VS [26]. An increase in striatal activity without feedback has also been reported during working memory and category learning, which supports the notion that the satisfaction of internal goals and incentives can drive striatal activity [27], [28]. In the category learning study, VS activity was, similar to the present data, correlated with confidence and, moreover, also with the prediction errors on confidence [27]. In the present recognition test, subjects either from the very beginning or rapidly develop the expectation that they will have only low confidence in their judgements. Therefore, the VS activity could represent, in addition to the subjective values of retrieval outcomes, positive prediction errors on the rare high confidence events.

The VS also plays a role in the detection of non-rewarding deviant and salient stimuli [7], [29]–[32]. However, the resulting alternative interpretation of the VS activity, i.e. that it may be related to the salience of the relatively few high confidence items in the overall low confidence context, seems unlikely given that the pleasantness ratings of the subjects parallel striatal activity.

Activity in parts of the VS was not only related to confidence but also to perceived oldness, i.e. the well documented retrieval success effect. Moreover, participants reported post experimentally that both, confidence and perceived oldness, satisfied their goals where confidence was the dominant cause for pleasantness. Together, this might imply that even in a context of high uncertainty the subjective value of detecting targets is higher than of detecting lures, i.e. that the subjective value of a given retrieval outcome is a function of both perceived oldness and confidence. Alternatively, the correlation of VS activity and pleasantness with both confidence and perceived oldness could also indicate two independent underlying processes. Recently, an elaborate review hypothesized three, non-mutually exclusive processes that potentially account for the striatal activity associated with retrieval success [4]. It will be discussed in the following whether the three process would also account equally well for the correlation with confidence, in particular for perceived new items.

According to the ‘Retrieval as Adaptive Encoding’-hypothesis, the VS modulates the re-encoding of items dependent on their subjective value, i.e. their behavioural relevance and the likelihood of their future utility [4]. The authors argue that the retrieval of an item in a given context signals a higher utility of that item, i.e. this item might be useful in the same context again. This re-encoding interpretation is supported by the involvement of the VS in encoding information beyond that associated with reward [33]–[35]. Moreover, it is known from the ‘testing effect’-literature that retrieval-attempts drive successful (re)-encoding, in particular for ‘old’-responses, which is amplified by confidence [36]. In agreement with this interpretation, a recent model suggests that dopaminergic neurotransmission in the ventral tegmental-hippocampal loop might underlie the re-encoding during retrieval [37]. The adaptive encoding hypothesis seems able to explain the confidence dependent activity for perceived old items in the present study. Moreover, re-encoding also of high confident rejected items in an uncertain context is consistent with this view since cognitive control determines which retrieval outcome is of high utility in a given situation, i.e. all high confidence items have a high utility in an uncertain context.

The ‘Reinforcement Learning and Adaptation of Cognitive Control at Retrieval’-hypothesis proposes that striatal activity is related to the evaluation of the employed retrieval strategies [4]. The VS codes expectations about the value of a retrieval strategy and computes a negative prediction error when a given stagey is ineffective, i.e. does not result in the desired retrieval outcome. This hypothesis is then also consistent with the correlation of VS activity with confidence for ‘old’- and ‘new’-responses in the present study. Since high confident rejections also have a high subjective value in this context, retrieval strategies that led to such an outcome would be reinforced by a positive prediction error. However, note that this positive prediction error over retrieval strategies differs conceptually from the prediction error over rare, pleasant, high confident decisions that were discussed earlier together with the category learning study [27].

The ‘Adaptive Gating of Working Memory to Control Retrieval’ hypothesis states that the striatum - though not necessarily ventral striatum - might be involved in selecting which representations to admit into working memory in order to increase the likelihood of a successful retrieval outcome and/or successful performance on the task overall. Thus, assuming that confidence reflects the strength of the decision that an item is old or new, then more confident decisions would have higher adaptive value for making a response. In other words, high confidence old or new are more likely to yield correct responses. Thus, striatal activation would track confidence in the task accordingly.

In conclusion, the three hypotheses account equally well for the correlation of VS activity with perceived oldness and with confidence, but only in a context which increases the subjective value of all types of high confidence retrieval outcomes. Therefore, it cannot be decided whether the three hypothesized processes contribute differently to the correlation of VS activity with perceived oldness and confidence. As a consequence, it cannot be inferred based on the hypotheses whether distinct processes in the VS might be the basis for the observed correlations with perceived oldness and confidence.

However, in reference to the question of whether the same or different processes in the VS correlate with confidence and perceived oldness, the exclusive masking descriptively showed a larger cluster in the bilateral VS to be correlated with confidence, a finding that was statistically confirmed for the right VS (Figure 2). This activity pattern was somehow paralleled by the post experimental reports of subjects, where most indicated that confidence was dominant for their pleasantness ratings followed by perceived oldness. Therefore, a potential contribution of different processes to the signal correlated with perceived oldness and confidence could be paralleled by a functional subdivision of the VS during recognition, especially since such a specialization has been suggested based on connectivity patterns [38].

The observed VS activity increase for retrieval success replicates many previous findings. It was hypothesized that this activity reflects the higher subjective value of hits than correct rejections in a standard recognition memory test, which was confirmed by the old/new effect in the pleasantness ratings of the present study [1], [8]. To test the subordinate hypothesis, it was moreover shown in the present experiment that the retrieval success effect in the VS does not differ significantly for true and false memories. On the contrary, using Deese–Roediger McDermott-paradigms, it has been shown that activity in other areas correlates with the factual memory status, i.e. is higher for hits than false alarms [39]. For example, greater activity for hits in the parahippocampus was explained by the reactivation of perceptual, or other encoding-related information, that is missing for false alarms. Based on these findings, the insensitivity of the VS to the factual status of a probe item in the current study provides evidence for the previous conclusion that VS activity is not triggered automatically by the outcome of retrieval, i.e. does not reflect the ‘retrieval-dependent’-account [8]. Perceived oldness during recognition has received much less attention than the retrieval success effect, presumably because of the high number of false alarms required for this type of analysis [20], [40]. Therefore, it cannot be determined based on the literature whether the correlation of VS activity with perceived oldness is a result of the unusually high rate of incorrect responses in the current paradigm or a general feature thereof.
